# Colchicine: the good, the bad, the ugly and how to minimize the risks

**DOI:** 10.1093/rheumatology/kead625

**Published:** 2023-11-29

**Authors:** Lisa K Stamp, Carl Horsley, Leanne Te Karu, Nicola Dalbeth, Murray Barclay

**Affiliations:** Department of Medicine, University of Otago, Christchurch, Christchurch, New Zealand; Critical Care Complex, Middlemore Hospital, Auckland, New Zealand; Faculty of Medicine, University of Auckland, Auckland, New Zealand; Faculty of Medicine, University of Auckland, Auckland, New Zealand; Department of Medicine, University of Otago, Christchurch, Christchurch, New Zealand; Department of Clinical Pharmacology, Te Whatu Ora, Waitaha Canterbury, New Zealand

**Keywords:** colchicine, overdose, toxicity, pharmacology, safety

## Abstract

Colchicine has an important role in managing various conditions, including gout, familial Mediterranean fever, amyloidosis, Behçet’s syndrome, recurrent pericarditis and calcium pyrophosphate deposition disease. The adverse effect profile of colchicine is well understood. However, due to its narrow therapeutic index, colchicine has been associated with overdose and fatalities. When ingested in toxic amounts, the mainstay of management is supportive care. Strategies to minimize the risk of colchicine poisoning can focus on three broad causes: unauthorized access, intentional overdose and inappropriate dosing. Culturally safe and appropriate education about storage and appropriate use of colchicine is essential to minimize the risk of overdose.

Rheumatology key messages

- Colchicine is an effective medication for a variety of conditions, including gout.
- When taken in excess, colchicine can cause serious and often fatal poisoning.
- Education about storage and appropriate use of colchicine is essential to mitigate the risk of overdose.

## Introduction

Colchicine is derived from two plants, *Colchicum autumnale* (autumn crocus, saffron) and *Gloriosa superba* (glory lily). It is used in the management of a variety of chronic conditions, including gout, FMF, amyloidosis, Behçet’s syndrome, recurrent pericarditis, calcium pyrophosphate (CPP) deposition diseases and Sweet’s syndrome. There is also emerging evidence for the use of colchicine in cardiovascular disease. However, when taken in excess, colchicine can cause serious and often fatal poisoning. It is therefore important that both healthcare providers and people being prescribed colchicine understand the risks and how it is safely stored and used. Herein, we review the pharmacology of colchicine, dosing, clinical features and management of colchicine poisoning and strategies to reduce the risk of overdose/poisoning.

## Pharmacology and mechanism of action

When ingested, standard oral doses of 0.6–1 mg of colchicine are rapidly absorbed from the gastrointestinal tract, with peak plasma concentrations occurring ≈1 h after ingestion [1]. However, peak concentrations occur later with higher doses (1.8 h with 1.8 mg and 4.5 h with 4.8 mg) [2], suggesting a saturable influx transporter for colchicine in the gut wall. The oral bioavailability of colchicine is ≈50% [1], the volume of distribution is 5–8 l/kg [3] and it has 40% protein binding to albumin [4]. Colchicine is a substrate for P-glycoprotein (P-gp) [5], which is present in the gut wall, kidneys, liver, and blood–brain barrier, with its primary function being to facilitate the export of xenobiotics such as colchicine out of the body to protect against toxicity. Colchicine is also metabolized by demethylation via cytochrome P4503A4, but <5% of clearance is by this route [1, 2, 6]. Around 10–20% is removed via renal excretion, and the majority of drug is eliminated as parent drug or metabolites in the faeces [2]. Following a single 1 mg dose, colchicine plasma half-life is ≈4.4 h in young healthy adults [2], but this increases to 26–31 h following multiple oral doses of 0.6 mg twice daily, consistent with enterohepatic recycling (and possibly some auto-inhibition of clearance) [2]. The half-life will be even longer in older people, those with renal or hepatic dysfunction or those who have ingested toxic amounts. In people with moderate–severe renal impairment (creatinine clearance 10–30 ml/min) colchicine clearance is halved and so the dose needs to be halved to reduce the risk of toxicity [7].

Colchicine accumulates in neutrophils, with the majority of its effects mediated through its ability to bind to tubulin monomers, thereby preventing the formation of heterodimers of microtubulin, which are involved in cell division, signal transduction, regulation of gene expression and migration [8]. Other anti-inflammatory actions of colchicine include inhibition of monosodium urate (MSU)-induced NLRP3 inflammasome activity in macrophages [9], alteration of adhesion molecule expression in endothelial cells and neutrophils [10] leading to a reduction in neutrophil adhesion and recruitment into inflamed joints and inhibition of a characteristic protein tyrosine phosphorylation pattern induced when neutrophils are exposed to MSU crystals [11].

A key Issue with colchicine is its narrow therapeutic index. Serum concentrations of colchicine relate poorly to efficacy since the anti-inflammatory effects are predominantly due to intracellular accumulation. Effective steady-state plasma concentrations have been reported to range from 0.5 to 3 μg/l, with toxic effects occurring at ≈3 μg/l [12]. Recent studies have shown that doses of 0.5 mg twice daily and 0.6 mg daily maintain sustained serum levels within the steady-state range in healthy individuals and individuals with mild–moderate renal impairment or concomitant use of most interacting medications [13].

## Important drug interactions

Due to the combination of limited oral availability and being a substrate for P-gp and CYP3A4, colchicine is subject to a range of significant drug interactions, especially with P-gp or CYP3A4 inhibitors. Concurrent use of CYP3A4-inhibiting drugs can result in a doubling of colchicine plasma concentration, and the use of P-gp inhibitors may quadruple colchicine concentrations [4]. Considering the low apparent percentage of colchicine that is metabolized by CYP3A4, it is likely that the interactions attributed to CYP3A4 are actually due, at least in part, to P-gp inhibition, as many CYP3A4 inhibitors are also P-gp inhibitors. Interacting drugs include diltiazem, verapamil, amiodarone, itraconazole, ketoconazole, fluconazole, ritonavir, saquinavir, other protease inhibitors, erythromycin, clarithromycin, ciclosporin and telmisartan [14]. Co-administration of verapamil, a potent P-gp and CYP3A4 inhibitor, has been shown to reduce the clearance of colchicine by ≈50%, thereby increasing plasma colchicine concentrations, indicating that the dose of colchicine should be reduced by ≈50% in individuals receiving concomitant verapamil [15].

## Clinical uses of colchicine and dosing

The indications and dosing of colchicine, both established and emerging, are outlined in Table 1. Lower doses are recommended for those with renal impairment and those receiving medications known to interact with colchicine (Table 2).

**Table 1.** Established and emerging indications and doses of colchicine

Disease		Dosing regimen	Duration of therapy	Reference
Established				
Gout	Gout flare treatment	1.0 mg stat then 0.5 mg 1 h later		16–18
	Gout flare prophylaxis when starting urate-lowering therapy	0.5/0.6 mg daily	Up to 6 months	19, 20
CPPD	Acute CPP crystal arthritis treatment	1.0 mg stat then 0.5 mg 1 h later		21
	Acute CPP crystal arthritis prophylaxis	0.5/0.6 mg daily	Long term	22; no clinical trial data
Acute and recurrent pericarditis		0.5-1.0 mg daily		23–25
FMF and other autoinflammatory syndromes		Up to 2.4 mg for adults	Long term	26, 27
Behçet’s syndrome		0.5–0.6 mg twice daily		28
Emerging				
Atrial fibrillation		0.5 twice daily		29, 30
Coronary artery disease		0.5 once to twice daily		29, 31

**Table 2.** Recommended dosing adjustment for colchicine [32]

		Gout flares	Prophylaxis against flare when starting urate-lowering therapy
General		1.2 mg stat followed by 0.6 mg 1 h later. Dose to be repeated no earlier than 3 days	0.6 mg once to twice daily
Renal impairment	Mild–moderate (CrCl >30–60 ml/min)	No dose adjustment required but closely monitor for adverse events	No dose adjustment required but closely monitor for adverse events
	Severe (CrCl <30 ml/min)	No dose reduction required, but do not repeat course within 2 weeks. If repeat courses required, consider alternative therapy	0.3 mg daily with any increase under close monitoring
	Dialysis	0.6 mg stat. Do not repeat more than once every 2 weeks	0.3 mg twice weekly
Hepatic impairment	Mild–moderate impairment	No dose reduction required	No dose reduction required
	Severe impairment	No dose reduction required, but do not repeat course within 2 weeks. If repeat courses required, consider alternative therapy	Consider dose reduction
CYP3A4	In patients with normal renal/hepatic function		
	Strong (e.g. clarithromycin, ketoconazole, itraconazole)	0.6 mg stat followed by 0.3 mg 1 h later. Dose to be repeated no earlier than 3 days	0.3 mg daily or every second day
	Moderate (e.g. diltiazem, verapamil, erythromycin)	1.2 mg single dose. Dose to be repeated no earlier than 3 days	0.3 mg once to twice daily
PGP inhibitors	In patients with normal renal/hepatic function		
	e.g. ciclosporin A	0.6 mg stat. Dose to be repeated no earlier than 3 days	0.3 mg daily or every second day

CrCl: creatinine clearance.

There is ample data that colchicine is effective in the management of gout. Colchicine is effective at preventing gout flares when commencing urate-lowering therapy [19, 20, 33, 34] as well as for the treatment of gout flares [16, 33]. It is essential to recognize that the older dosing regimen for treating gout flares, namely 1.2 mg stat followed by 0.6 mg hourly until the flare resolves or diarrhoea occurs, is no longer recommended due to the high rates of gastrointestinal toxicity and the evidence that lower doses of colchicine are effective without the associated gastrointestinal effects [16, 17]. In rheumatology practice, colchicine is also widely used in the treatment of CPP deposition disease, particularly to prevent and treat acute CPP crystal arthritis [21, 22], as well as in the treatment of Behçet’s syndrome, FMF and other systemic autoinflammatory syndromes [35–37]. While colchicine has been used for some time in acute and recurrent pericarditis, more recently its beneficial effects have been observed in other cardiovascular diseases, including postoperative atrial fibrillation and coronary artery disease. The evidence for its use in cardiovascular disease has recently been reviewed by Deftereos *et al.* [29].

The adverse effects of low-dose colchicine are also well known. The most common adverse effect is diarrhoea, which settles on cessation of colchicine. Small increases in alanine transaminase and creatine kinase are common with colchicine treatment over 2–4 years [38]. Long-term colchicine therapy has also been associated with rare cases of neuromyotoxicity and rhabdomyolysis [39, 40]. There is no evidence that long-term colchicine increases the risk of kidney disease [38], cancer, sepsis or cytopaenias [41].

## Colchicine poisoning

**Figure 1.**
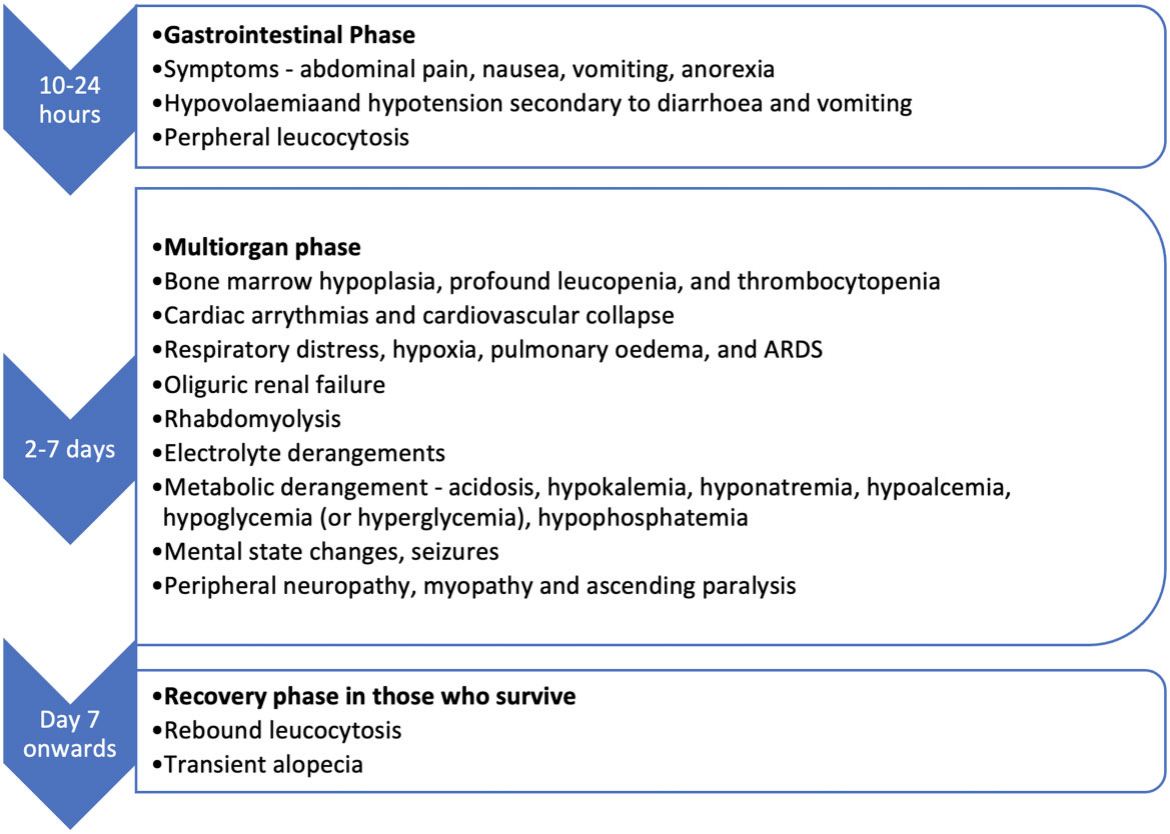
Clinical features of the three phases of colchicine poisoning

As noted above, colchicine has a narrow therapeutic index. Colchicine has been associated with fatalities with doses as low as 3 mg [42]. Colchicine doses >0.5 mg/kg, and especially >0.8 mg/kg, are generally fatal. The clinical features of colchicine poisoning are a consequence of the arrest of cellular mitosis. Typically, three clinical phases are described: early (10–24 h), mid (2–7 days) and late (beyond day 7) (Fig. 1). In a series of 21 cases of colchicine poisoning, the mean age was 25.48 years (s.d. 12.65), 61.9% were female, 85.7% were an intentional overdose and the mean ingested colchicine dose was 30.25 mg (s.d. 21.09). It is important to note that 14.3% died. Factors associated with poor prognosis included nausea and vomiting, abdominal pain, abdominal tenderness, disseminated intravascular coagulation (DIC) and requirement for intubation and mechanical ventilation. Blood pressure, serum glucose, calcium and partial pressure of carbon dioxide were significantly lower while aspartate aminotransferase, alkaline phosphatase, prothrombin time and activated partial thromboplastin time measures were considerably higher in non-survivors [43]. Cardiac failure is also associated with a poor prognosis. Table 3 provides details of additional cases and their outcomes identified in the literature. There is a lack of large observational studies on the toxicity effect of colchicine according to different comorbidities or concomitant use of other medications. Future research should include using large electronic medical record databases to generate evidence-based findings to guide clinical practice.

**Table 3.** Case reports of colchicine overdose

Reference	Age and gender	Colchicine dose	Key clinical symptoms	Treatment	Outcome
Aghabiklooei *et al.* [44]	10-year-old male	30 mg	Nausea and vomiting followed by multi-organ failure, cardiac	Gastric lavage, charcoal, sorbitol	Death at ≈48 h post-ingestion
	37-year-old female	38 mg	Multi-organ failure	Charcoal, sorbitol	Death at 36 h post-ingestion
	25-year-old female	25 mg	Thrombocytopaenia	Activated charcoal, G-CSF	Survived
Arroyo *et al.* [45]	48-year-old male^a^	18 mg	Intractable nausea and vomiting followed by multi-organ failure and encephalopathy, TEN-like reaction	G-CSF, dialysis	Death on day 9
Ataş *et al.* [46]	12-month-old female	3 mg (0.37 mg/kg)	Coma	Gastric lavage, charcoal	Death at 13 h
	2-year-old boy	6 mg (0.46 mg/kg)	Vomiting	Gastric lavage, charcoal	Survived
	27-month-old female	10 mg (12 mg/kg)	Vomiting and diarrhoea followed by reduced consciousness	Gastric lavage, charcoal	Death at 6 h
	3.5-year-old female	25 mg (1.725 mg/kg)		Gastric lavage, charcoal	Survived
Baldwin *et al.* [47]	29-year-old male	Snorted ≈200 mg	Nausea and vomiting	Supportive care	Survived
Baud *et al.* [48]	25-year-old female	60 mg (0.96 mg/kg)	Hypotension	Colchicine-specific Fab, fluid replacement	Survived
Blackham *et al.* [49]	34-year-old female	Unknown	Mild gastrointestinal symptoms with rapid progression to fulminant hepatic failure and multiple organ dysfunction	Supportive care	Survived
Caplan *et al.* [50]	39-year-old female	≈30 mg colchicine, concentration 250 µg/l at 2 h post-dose	Drowsy, dizzy and began vomiting and diarrhoea then multi-organ failure		Death at 40 h
Critchley *et al.* [51]	43-year-old female	25–30 mg (≈0.6 mg/kg)	Abdominal pain, nausea and vomiting, pancytopenia, alopecia	Supportive care, G-CSF	Survived
Davies *et al.* [52]	28-year-old male	750 mg colchicine powder	Nausea followed by progressive renal failure, hepatocellular damage and coagulopathy		Death at 48 h
Deng *et al.* [53]	19-year-old	80 mg concentration 5 ng/ml at day 12	Multi-organ dysfunction syndrome and sudden cardiac arrest	Continuous renal replacement therapy, haemoperfusion and therapeutic plasma exchange	Death at day 4
	70-year-old female^a^	5 mg		Abdominal pain, diarrhoea and vomiting	Death
Dickinson and Junja [54]	37-year-old female	15 mg	Diarrhoea, followed by multi-organ failure		Death at day 4
Dodds *et al.* [55]	25-year-old female	40 mg	Nausea, vomiting and abdominal pain, renal failure, DIC, GI bleeding		Survived
Essame *et al.* [56]	19-year-old male	24 mg (0.4 mg/kg)	Multi-organ dysfunction, pancytopenia	Supportive care	Survived
Folpini and Furfori [57]	24-year-old female	>50 mg	Severe abdominal pain, nausea, vomiting, pancytopenia, alopecia	Gastric lavage, charcoal, G-CSF	Survived
Fu *et al.* [58]	56-year-old male^a^	12 mg (0.17 mg/kg)	Severe abdominal pain, nausea, frequent diarrhoea and vomiting rapidly progressed to abdominal pain, respiratory insufficiency, circulatory failure, acute liver failure, acute renal failure and coagulopathy	Gastric lavage	Death at ≈day 7
Güven *et al.* [59]	4-year-old female	45–48 mg (1.3–1.5 mg/kg)	Abdominal pain, diarrhoea, vomiting, pancytopenia and alopecia	G-CSF	Survived
Hirayama *et al.* [60]	18-year-old female	15 mg (0.2 mg/kg)	Abdominal pain followed by respiratory distress syndrome and cardiac shock, myelosuppression	G-CSF	Survived
Hobson and Rankin [61]	15-year-old male	18 mg (≈0.4 mg/kg)	Vomiting and diarrhoea followed by multi-organ failure	Supportive care	Death on day 5
Huang *et al.* [62]	48-year-old male^a^	>10 mg	Oligo/anuria and diarrhoea	Supportive care	Survived
Iosfina *et al.* [63]	47-year-old female	90 mg	Abdominal pain followed by multi-organ failure	Activated charcoal, N-acetylcysteine and supportive care	Survived
Jayaprakash *et al.* [64]	39-year-old male^a^	18–24 mg	Diarrhoea and vomiting followed by multi-organ failure	Activated charcoal	Death on day 3
	15-year-old female	30 mg	Diarrhoea and vomiting followed by multi-organ failure	Supportive care	Death at 2.2 days
	56-year-old male	24 mg	Diarrhoea and vomiting followed by multi-organ failure	Activated charcoal	Death
	59-year-old male	18 mg	Shock	Supportive care	Death within h
	15-year-old male	18 mg	Diarrhoea and vomiting followed by multi-organ failure	Supportive care	Death on day 4
	19-year-old male	Unknown	Vomiting followed by multi-organ failure	Supportive care	Death on day 4
	20-year-old male	40 mg	Abdominal pain and diarrhoea followed by multi-organ failure	Activated charcoal	Death at 96 h
	46-year-old male	Unknown	Hypotensive and metabolic acidosis	Supportive care	Survived
Jouffroy *et al.* [65]	51-year-old male	17 mg	Multi-organ failure	Extracorporeal life support, dialysis	Survived
Katz *et al.* [66]	19-year-old male	30–36 mg	Fever and pancytopenia, rhabdomyolysis	Activated charcoal, G-CSF	Survived
Kocak *et al.* [67]	26-year-old female	27.5 mg	Diarrhoea, pancytopenia, alopecia	Gastric lavage, charcoal	Survived
Lev *et al.* [68]	18-year-old female	18 mg (≈0.4 mg/kg)	Vomiting and diarrhoea, pancytopenia, respiratory distress	N-acetylcysteine, G-CSF	Survived
Little *et al.* [69]	39-year-old female	25 mg (0.28 mg/kg)	Nausea, vomiting, abdominal pain, multiorgan failure	Activated charcoal, rifampin 600 mg i.v. in an attempt to induce the metabolism of colchicine	Death at 2 days
Maxwell *et al.* [70]	41-year-old male^a^	27 mg over 24–48 h	Abdominal pain, diarrhoea, vomiting, EMD cardiac arrest		Death at ≈36 h
Montiel *et al.* [71]	33-year-old woman	20 mg (≈0.3 mg/kg), ibuprofen (8 g), diclofenac (1 g), atorvastatin (100 mg) and furosemide (400 mg)	Diarrhoea, diffuse bleeding complications, the worsening of hypoxaemia and the development of an intestinal ileus, generalized seizures, pancytopenia and severe hypertriglyceridaemia	Supportive care	Death on day 14
Schaffer *et al.* [72]	37-year-old male	36 mg colchicine, concentrations 5.1 ng/ml (30 h post-ingestion) and 12 ng/ml (40 h post-ingestion)	Multi-organ failure including coagulopathy, respiratory failure, neuropathy, renal failure, pancytopenia and heart failure	N-acetylcysteine, dialysis	Survived
Stringfellow *et al.* [73]	57-year-old male^a^	10 mg	Abdominal pain, nausea and vomiting	i.v. glucose, dopamine and supportive care	Died at 26 h
Trebach *et al.* [74]	13-year-old boy	Unclear colchicine concentration day 1 12 ng/ml	Nausea, vomiting, abdominal pain and diarrhoea followed by altered mental status, acute hypoxic respiratory failure and cardiogenic shock	ECMO and continuous kidney replacement therapy, exchange transfusion	Death at day 8
van Heyningen and Watson [75]	21-year-old woman	25 mg (0.25 mg/kg)	Nausea, vomiting then multi-organ failure	Activated charcoal	Death at day 3
Wacker *et al.* [76]	74-year-old female	Ingestion of a pizza covered with *Colchicum autumnale* leafs	Cardiogenic shock	Activated charcoal, ECMO, dialysis and plasma exchange—total of 7.6 mg colchicine was removed	Survived
Yamazaki *et al.* [77]	38-year-old male^a^	15 mg (0.2 mg/kg)	Nausea, vomiting, diarrhoea, hematemesis and multi-organ failure	G-CSF	Survived
Zhong *et al.* [78]	19-year-old woman	40 mg (0.9 mg/kg)	Abdominal pain followed by multisystem failure including renal, respiratory, circulatory and digestive	Dialysis supportive care	Survived

ECMO: Extracorporeal membrane oxygenation; EMD: electromechanical dissociation.

a Indicates those cases where colchicine was taken in an attempt to relieve symptoms of gout.

## Management of colchicine poisoning

There are currently no specific or effective treatments for colchicine poisoning, meaning the mainstay is supportive care only. Anyone with a known or suspected colchicine overdose should be admitted for observation for the first 24 h post-ingestion. If no symptoms or signs have developed within this period, they are unlikely to occur. Activated charcoal or gastric lavage may be useful for individuals who present early after ingestion to prevent further gastrointestinal absorption, but this can be complicated by the presence of vomiting.

Several mechanisms have been tried in an attempt to remove excess colchicine. Treatment with haemodialysis and plasma exchange are complicated by colchicine’s short half-life and ability to bind to tissues. The rationale for plasma exchange is that colchicine is ≈40–50% protein bound at therapeutic doses. The data are conflicting as to the benefit, with a case report of plasma exchange revealing removal of only 0.01% of the ingested dose, an amount that is unlikely to influence clinical outcome [76], while other reports have suggested potential benefit [72]. Haemodialysis is indicated to support significant renal impairment rather than as a mechanism for the removal of colchicine.

Several animal models have shown that colchicine-specific antigen-binding fragments can reverse the effects of colchicine [79–82]. Treatment of colchicine poisoning with colchicine-specific fragment antigen-binding (Fab) has been reported [48]. In this case, the colchicine Fab infusion led to a dramatic increase in plasma colchicine concentrations, indicating substantial amounts of the drug were removed from peripheral sites and redistributed into the extracellular space, where they were retained due to high-affinity binding to the Fab. There was a 6-fold increase in the urinary excretion of colchicine [48, 81]. Lack of commercially available colchicine-specific Fab precludes further clinical trials aimed at determining the efficacy and safety of Fab in colchicine poisoning. N-acetylcysteine has been used in an attempt to reduce colchicine-induced oxidative stress [63, 68], although the impact on clinical outcomes is unclear.

## Strategies to minimize the risk of poisoning

The New Zealand National Poisons Centre received 56 cases of poisoning from 1 January 2016 to 14 January 2021; 43% were children 1–4 years of age gaining unauthorized access to the medication, 21% were intentional overdose and 35% were thought to be due to a misunderstanding of how to take colchicine appropriately for gout flares or increasing the dose to obtain additional symptom relief [83]. Strategies to minimize the risk of colchicine poisoning can focus on these three core causes: unauthorized access, intentional overdose and inappropriate dosing for gout flares.

While any medication can be accessed in an unauthorized manner or for intentional overdose, it is the uniquely toxic properties of colchicine that result in its unintended fatal consequences. It is unlikely that children and many of those taking colchicine understand these unique properties and the high risk of death. Strategies to minimize the risk of unauthorized access can be grouped into three broad areas: modifying how the medication is stored or accessed, raising awareness leading to modified behaviour through education and regulations that modify individual behaviour (Table 4). Child-proof packaging was estimated to reduce the child mortality rate from accidental overdose of prescription medications by 45% between 1974 and 1992 [84]. However, child-proof packaging is not infallible and people need to be educated about ensuring proper closure after each use, not transferring medications from the original container [85] and safe storage up away and out of sight. Regulating the number of colchicine tablets that can be dispensed at one time is a form of enforcement. However, given that in some individuals even a small number of tablets can be toxic and many of the conditions for which colchicine is prescribed require a continuous supply, such a strategy seems unlikely to be effective.

**Table 4.** Strategies to minimize the risk of colchicine overdose

Engineering: modification of product storage and access	Education: individual behaviour modification	Enforcement: regulation to modify individual behaviour
Pharmacy labels and handouts in appropriate languages	Clinicians deliver culturally appropriate and safe education. Non-regulated workforce engaged with ensuring messaging has been received and assimilated	Regulation—authorities/licensing bodies ensure robust accreditation of clinicians to deliver culturally safe care
Keep medication up, away and out of sight	Education about dangers of medication and requirement to keep up and away	Limit number of individual tablets that can be dispensed at one time
Child-resistant packaging—reliance on patients or caregivers to properly cap and safely store medications immediately after every use	Keep in original packaging. Must replace cap securely after use	
Unit dose packaging—no need to resecure safety barriers of unit-dose packaging	Appropriate dosing for the condition and when to seek medical attention if colchicine not providing sufficient benefit	

In addition to unauthorized access, inappropriate colchicine dosing for gout flares is the other situation that should be avoidable with education about the risks and benefits and the correct dosing of colchicine. People with gout report increasing the dose of colchicine in an attempt to control gout flares [86]. This has risks and it is important that people with gout know to seek medical attention if a gout flare fails to settle as expected with colchicine rather than simply taking more. Limiting the number of colchicine tablets dispensed for people with gout would provide yet another barrier to appropriate gout treatment and may compound existing health inequity [87]. Thus the key strategy should be raising awareness about safe use.

## Culturally safe care

Gout disproportionately affects indigenous Māori as well as Pacific peoples resident in Aotearoa/New Zealand. Evidence shows that Māori and Pacific peoples are more likely to be exposed to colchicine toxicity. Of the 56 cases of colchicine poisoning from 1 January 2016 to 14 January 2021 reported to the New Zealand National Poisons Centre, 34% were Māori or Pacific peoples and 13% were New Zealand European, with the ethnicity of the remaining cases unknown [83]. Education on colchicine and its potential benefits and risks must be delivered in an understandable manner under an umbrella of culturally appropriate and safe care.

## Conclusion

Colchicine is an effective medication for many conditions but has a narrow therapeutic index. Colchicine in overdose is frequently fatal and there is no specific therapy other than supportive care. Appropriate, effective education about storage and appropriate use of colchicine is essential to eliminate the risk of overdose.

## Data Availability

No new data were generated or analysed in support of this research.
